# Reduced Orofacial Health and Esthetics, but Perception of Good Generic Health and Quality of Life in Middle-Aged Patients with UCLP

**DOI:** 10.1177/10556656261444374

**Published:** 2026-04-20

**Authors:** Asgjerd Litleré Moi, Hallvard Andreas Vindenes

**Affiliations:** 1 Department of Health and Caring Sciences, 1657 Western Norway University of Applied Sciences, Bergen, Norway; 2 Department of Plastic, Hand and Reconstructive Surgery, 60498 Haukeland University Hospital, Bergen, Norway; 3 Faculty of Health Sciences, Institute for Nursing, VID Specialized University, Bergen, Norway; 4 Department of Clinical Dentistry, 1658 University of Bergen, Bergen, Norway

**Keywords:** UCLP, adults, health, quality of life, coping, self-esteem, social support, work

## Abstract

**Objective:**

Describe self-perceived health and quality of life in middle-aged adults with unilateral complete cleft lip and palate (UCLP).

**Design:**

Questionnaire study.

**Setting:**

Multidisciplinary care at a Norwegian university hospital and department of clinical dentistry.

**Participants:**

Twenty-one adults (37–44 years) with UCLP, operated on by the same surgeon and followed to early adulthood according to a standardized protocol.

**Outcomes:**

Oral-specific health, orofacial esthetics, generic health and overall quality of life. Independent variables: Coping, self-esteem, perceived social support and socio-demography.

**Results:**

Participants reported significantly poorer oral-specific health and lower satisfaction with orofacial esthetics than population norms. Functional limitations and difficulties related to speech and eating were most prominent. Regarding appearance, they were least satisfied with facial profile, mouth, and visible teeth. Generic health scores were comparable to norms; however, despite persistent orofacial concerns, participants reported significantly higher overall quality of life than the general population. Participants reported low avoidant coping, good self-esteem, and high perceived social support. Better oral-specific health and lower satisfaction with orofacial appearance were associated with lower avoidant coping and higher social support. Better overall quality of life was associated with being married/cohabiting, low avoidant coping, and high social support. Unemployment was linked to poorer outcomes across all health and quality of life dimensions.

**Conclusions:**

Despite persistent oral-facial health and appearance concerns, middle-aged adults with UCLP reported good generic health and high overall quality of life. Psychosocial resources, partnership and employment may buffer the impact of orofacial difficulties, underscoring the value of holistic, individualized, lifelong, and multidisciplinary follow-up.

## Introduction

Cleft lip and/or palate (CL/P) is a birth defect that requires surgical treatment and follow-up from infancy through childhood, adolescence and into young adulthood. The primary focus of treatment has traditionally been optimal orofacial functioning and esthetic outcomes, and the emphasis of treatment has been directed towards the disruption of facial growth, dental-status, speech, hearing and appearance.^1^ Standardized protocols for surgery and multidisciplinary follow up, as well as centralized care provision for patients with CL/P have improved the quality of care.^2^ Despite the heavy combined burden of the various interventions, there are several reports demonstrating high levels of patient satisfaction,^3–5^ but also reports describing the feeling of relief when discharged from the standard follow-up program as young adults.^5^

However, for a substantial fraction of CL/P patients, demand for multidisciplinary treatment may persist into adulthood, and new needs may also arise long after follow-up, according to the standard protocol, has stopped.^3,5–7^ Consistently, it is well documented that having CL/P significantly increases the use of hospital services throughout the lifespan.^8^ The challenges of adult CL/P patients may be physical health problems related to speech, hearing, breathing, eating, and drinking, but also mental health conditions and psychosocial challenges and concerns regarding appearance, uncertainty around having family and close friends, as well as occupational issues.^3,5–7^ Several studies suggest that there may be significant differences in the long-term outcome of CL/P patients, both related to type of cleft, organization and procedures of treatment and follow-up, as well as socio-demography.^9–13^ Despite these interindividual differences, the long-term problems seem to be interrelated and may affect most aspects of life, suggesting unmet needs that outreach the adult CL/P patients’ potential medical problems.^3,5,14,15^ Further understanding and assessment of these needs are important for being able to devise healthcare practices and policies that address them.

It has been claimed that a patient's evaluation of having CL/P and how it affects orofacial appearance may be more important in determining well-being than the traditional medical focus on professional evaluation of function and esthetics.^16^ Assessments of patient reported outcomes add significantly to the understanding of the consequences of an illness, and the various aspects of treatment and follow up provided, and measurements of self-perceived health and quality of life are today recommended as an important quality indicator in health care delivery.^17^ The scientific literature on patient reported outcomes in the adult CL/P population is increasing but still limited, and data obtained using validated instruments allowing for the comparison with general population norms or reference data are scarce. Moreover, the terminologies and questionnaires used have so far in most cases not strictly allowed for the discrimination between self-perceived health and overall quality of life. To overcome this in the present study, the conceptual model of health-relate quality of life described by Wilson and Cleary guided the selection of a comprehensive set of standardized questionnaires that together with socio-demographic and selected clinical parameters allowed for the collection of data on disease-specific symptoms, functioning, evaluation of own health and overall quality of life, as well as selected aspects of the individuals and the environment that may influence health and quality of life (Figure 1).^18^

**Figure 1.**
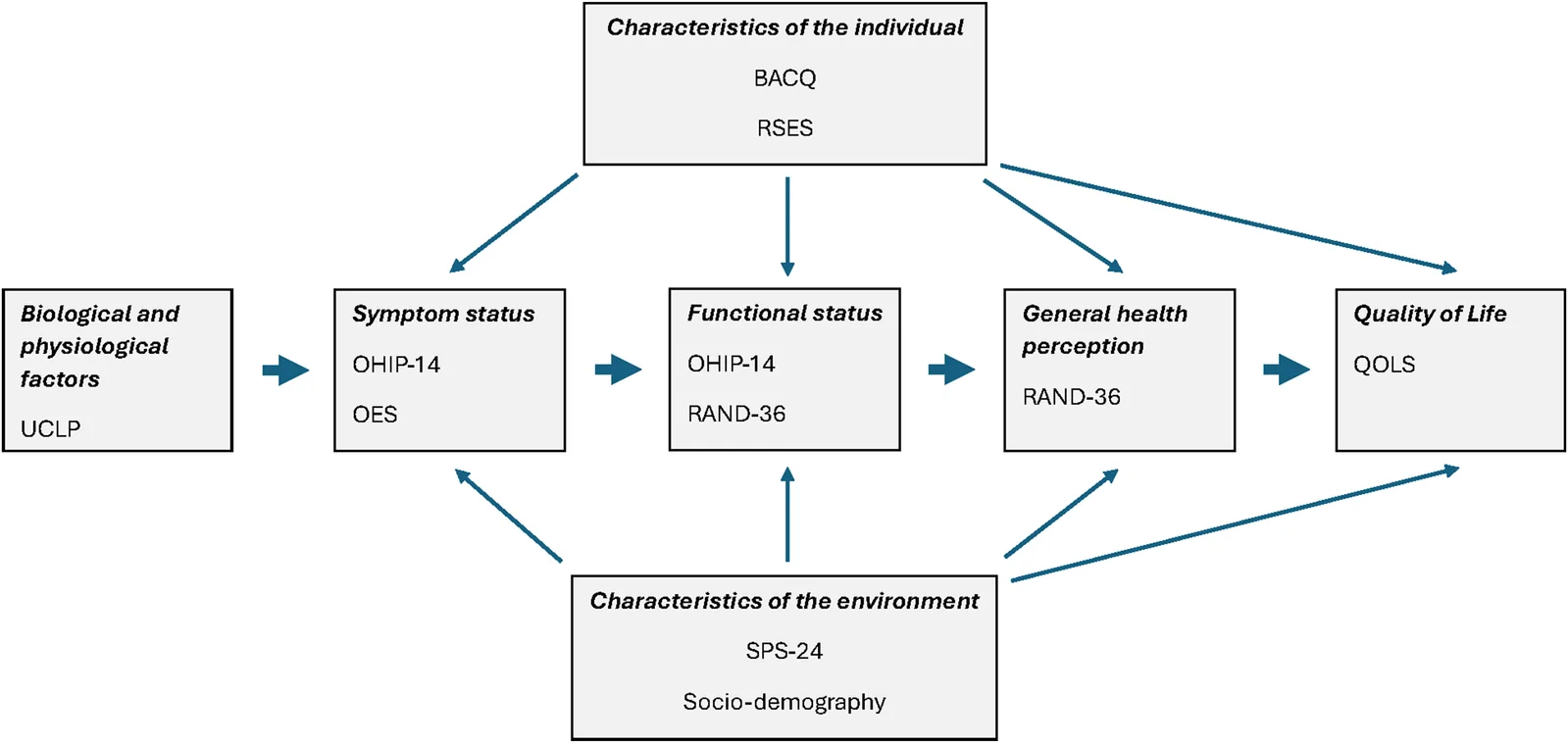
Model oyf health-related quality of life adapted from Wilson and Cleary showing the questionnaires covering disease-specific health (symptoms and function related to UCLP), general health and overall quality of life, as well as characteristics of the participants and their environment.^18^ UCLP: unilateral complete cleft lip and palate; OHIP-14: the oral health impact profile-14; OES: the orofacial esthetic scale; RAND-36: the RAND 36-item short health survey; QOLS: the quality of life scale; BACQ: the brief approach/avoidance coping questionnaire; RSES: the Rosenberg self-esteem scale; SPS-24: the social provision scale-24.

Specifically, the present study aimed to describe self-reported health and overall quality of life, as well as possible associated factors in a selected cohort of middle-aged patients with unilateral complete cleft lip and palate (UCLP) who all had been operated on by the same surgeon and received multidisciplinary follow up by the same team according to a standardized protocol.

## Materials and Methods

### Study Design

This explorative, descriptive questionnaire study on health and quality of life of middle-aged adults born with UCLP was performed as part of a multi-disciplinary follow-up to evaluate the overall long-term outcome of being born with and treated for this condition. The clinical evaluations included consultations by a plastic surgeon, an orthodontist, an otorhinolaryngologist and a speech therapist. Moreover, the patients were interviewed on their subjective experience of growing up and living with UCLP. The results of the interview study have been reported earlier.^5^

### Participants and Setting

A cohort of 30 adults that were treated for non-syndromic UCLP from 1973 to 1979 by the multi-disciplinary CL/P team in Bergen, Norway was invited by mail to participate. They all had their first surgical treatment at the age of three months, and closure of the palate at the age of 2 years. The patients were operated on according to the same procedure by the same surgeon, and the protocol and the initial data on their treatment and follow-up were reported in 2009.^19^

In total, 21 adults took part in this study, as three declined participation, and we were unable to locate six. The sample consisted of 12 men and nine women, and their age was 40.8 ± 2.0 (mean ± SD) years. Six of the participants reported slight asthma, atopic eczema, or migraine.

### Data Collection

The participants received a booklet containing seven standardized questionnaires, as well as 20 questions on socio-demography and health including age, gender, education, civil status, number of children, cohabitation, height and weight, comorbidity, problems with teeth, use of tobacco, and work status. They answered the questionnaires the same day as they took part in the multi-disciplinary follow-up consultations.

Publicly available socio-demographic data for people of the Norwegian general population who were 40 years of age at the time of the data collection of this study were obtained from Statistics Norway. Data on civil status, education, and paid work were obtained from StatBank Norway tables 06831^20^, 08921^21^, and 06445,^22^ respectively. Data on people having children were obtained from Statistics Norway's Statistics on Births.^23^

The standardized questionnaires were chosen according to the model of health-related quality of life described by Wilson and Cleary to cover outcomes of health (both disease-specific and generic health) and overall quality of life, as well as factors related to the participants as persons and their environment (Figure 1).^18^

### Measures of Health and Overall Quality of Life Outcome

#### Disease-Specific Health

1. *The Oral Health Impact Profile-14* (OHIP-14) assesses oral-specific health status as people's perception of the impact of oral conditions on their well-being.^24^ The questionnaire comprises 14 items distributed in 7 domains: Functional limitations, Physical pain, Psychological discomfort, Physical disability, Psychological disability, Social disability, and Handicap. The answers are given on a 5-point scale (never = 0, hardly ever = 1, occasionally = 2, fairly often = 3, and very often = 4). The results are given as domain scores and a total sum score. A lower score represents better oral health. OHIP-14 norm data from a representative sample of Norwegian adults were used as reference values.^25^
2. *The Orofacial Esthetic Scale* (OES) was used to assess the participants’ self-perceived orofacial appearance.^26^ The questionnaire comprises 8 items on facial appearance, facial profile, mouth, alignment of teeth, shape of teeth, color of teeth, appearance of gums, as well as a general orofacial esthetic assessment.^26^ The answers are given on a 0–10 numeric rating scale (from 0: Very dissatisfied to 10: Very satisfied). The results are reported as item scores and a total sum score of the first seven items.^26^ A higher score represents better perceived orofacial esthetics. Since Norwegian OES norms have so far not been established, OES norm data from a representative sample of Swedish adults were used as reference values.^27^

### Generic Health

*The RAND 36-item short health survey* (RAND-36) was used to assess generic health.^28,29^ The RAND-36 comprises 36 items addressing eight different health concepts, with the domains physical functioning, role limitations due to physical health, bodily pain, general health perceptions, vitality (energy/fatigue), social functioning, role limitations due to emotional problems and mental health. The answers in this study were transformed according to recommendations into scale scores 0–100 for each of the RAND-36 domains, where higher scores represent better self-perceived generic health status.^30^ RAND-36 norm data from a representative sample of Norwegian adults were used as reference values.^31^

### Overall Quality of Life

*The Quality of Life Scale* (QOLS) was used to assess the participants’ overall quality of life^.^^32^ The questionnaire consists of 16 items asking for degree of satisfaction with important aspects of people's lives. The answers are given on a 1–7 numeric rating scale (from 1: Very dissatisfied to 7: Very satisfied). The results are given as a sum score, and higher scores represent better overall quality of life. QOLS norm data from a representative sample of Norwegian adults were used as reference values.^33^

### Measures of Personal Factors Related to Health and Quality of Life

1. *The Brief Approach/Avoidance Coping Questionnaire* (BACQ) was used to assess the participants’ self-perceived coping.^34^ The BACQ is a 12-item questionnaire that assesses general coping strategies of approach and avoidance, as well as socioemotional, cognitive, and action-related coping. The questionnaire has been constructed and validated in a Norwegian sample of students and general practice patients showing satisfactory psychometric properties.^34^ The domain structure includes Approach (BACQ-A; items 1–6), Diversion (BACQ-D; items 7–9), and Withdrawal and Resignation (BACQ-WR; items 10–12).^34,35^ Answers are given on a 1-5 numeric scale (from 1: Disagree completely to 5: Agree completely). The results are reported as domain and total sum scores. When calculating the total sum score, the item scores of the domains BACQ-D and BACQ-WR are reversed. Hence, higher total sum score indicates more approach-oriented and less avoidant-oriented coping strategies. Since Norwegian BACQ norms have so far not been established, norm data from a representative sample of Danish adults were used for comparison.^35^
2. *The Rosenberg Self-Esteem Scale* (RSES) was used to assess the participants’ global self-esteem.^36^ The questionnaire contains 10 items with statements expressing positive and negative self-esteem. The answers are given on a 1-4 numeric rating scale (from 1: Strongly disagree to 4: Strongly agree). When calculating the total sum score, the item scores on negative self-esteem (items 2, 4, 6, 8 and 10) are reversed. Hence, higher total sum scores represent better global self-esteem. Since Norwegian RSES norms have so far not been established, norm data from a representative sample of US adults were used for comparison.^37^

### Measure of Environmental Factors Related to Health and Quality of Life

*The Social Provision Scale (SPS-24)* was used to assess the participants’ self-perceived availability of social support.^38^ The questionnaire contains 24 items arranged in six domains: Guidance, Reassurance, Social Integration, Attachment, Nurturance, and Reliable Alliance. The results are reported by domain or as a total sum score. The answers are given on a 1–4 numeric rating scale (from 1: Strongly agree to 4: Strongly disagree). Higher scores indicate better perceived availability of social support. Since Norwegian SPS-24 norms have not been established, data from a representative sample of Canadian adults were used for comparison.^39^

## Data Analysis

Descriptive data were presented as numbers and percentages for categorical variables and means and standard deviations for continuous variables. Welch's unequal-variances two-sample t-test was used to compare participant values with values from the general population norms. Bivariate associations between socio-demographic factors, personal factors (BACQ and RSES) or environmental factors (SPS-24) and oral-specific health (OHIP-14), orofacial appearance (OES), generic health (RAND-36) and overall global quality of life (QOLS) were performed using Spearman's rank correlation. IBM SPSS Statistics 30.0.0.0 and Microsoft Excel were used for the analysis.

### Ethics and Approvals

The study was conducted according to the Helsinki declaration.^40^ All participants gave written consent after having received an invitation letter and information including the study purpose, contact information, voluntary participation, and the fact that they could withdraw at any time before publication. The study was approved by the Regional Committee for Medical and Health Research Ethics (REC West; p.no. 2016/269).

## Results

Sixteen of the participants were married or cohabitating (ie, 76% compared to about 75% of 40-year-old people in the general population), 18 had children (ie, 86% compared to about 77% of 40-year-old people in the general population) and 15 had education at college or university level (ie, 71% compared to about 44% of 40-year-old people in the general population). Fifteen participants had paid work (ie, 71% compared to about 82% of 40-year-old people in the general population), whereas six were unemployed, of which three received social security money, two were temporarily out of work and one attended an occupational counseling program.

The patients with UCLP had significantly higher OHIP-14 total sum score [8.1 (7.3) (mean (SD)] than the Norwegian general population norms [4.1 (6.2) (mean (SD)], demonstrating overall poorer oral-specific health in the patient group (Table 1). Consistently, the UCLP scores of 5 of 7 OHIP-14 domains (ie, Functional limitations, Psychological discomfort, Physical disability, Psychological disability and Social disability) were significantly higher than the norms. In addition, the largest differences between the CLP group and the general Norwegian population on item level were related to difficulties pronouncing words, unsatisfactory diet, irritation and embarrassment caused by their orofacial condition (Table 2).

**Table 1.** Oral-Specific Health (OHIP-14), Orofacial Esthetics (OES), Generic Health (RAND-36) and Overall Quality of Life (QOLS) in Middle-Aged Patients with UCLP.

Measure	Total Sum Scores and Domain Scores (possible score variation)	UCLP		Norm Or Reference Group	
		mean (SD)	median (1. quartile – 3. quartile)	mean (SD)	p-value^e^
**OHIP-14**	Total score (0–56)	8.1 (7.3)	7.0 (3.0–11.0)	4.1 (6.2)^a^	0.021^f^
	Functional limitations (0–8)	1.3 (1.3)	1.0 (0.0–2.0)	0.1 (0.4)^a^	< 0.001
	Physical pain (0–8)	1.0 (1.1)	1.0 (0.0–2.0)	0.7 (1.0)^a^	0.227
	Psychological discomfort (0–8)	1.6 (1.9)	1.0 (0.0–2.0)	0.5 (0.9)^a^	0.015^f^
	Physical disability (0–8)	1.3 (1.7)	1.0 (0.0–2.5)	0.1 (0.5)^a^	0.004^g^
	Psychological disability (0–8)	1.5 (1.7)	1.0 (0.0–2.0)	0.3 (0.7)^a^	0.004^g^
	Social disability (0–8)	0.8 (1.0)	0.0 (0.0–1.5)	0.1 (0.4)^a^	0.004^g^
	Handicap (0–8)	0.7 (1.2)	0 0 (0.0–1.0)	0.2 (0.5)^a^	0.071
**OES**	Total score (0–70)	40.9 (12.6)	44.0 (30.5–51.5)	50.3 (15.6)^b^	0.002^g^
	Face (0–10)	6.3 (1.7)	5.0 (5.0–8.0)	7.6 (2.4)^b^	0.002^g^
	Profile (0–10)	5.8 (1.7)	5.0 (5.0–7.5)	7.5 (2.5)^b^	< 0.001
	Mouth (0–10)	5.4 (2.1)	5.0 (4.0–7.0)	7.2 (2.7)^b^	< 0.001
	Alignment teeth (0–10)	5.6 (2.4)	6.0 (3.5–7.5)	6.9 (2.9)^b^	0.023^f^
	Shape teeth (0–10)	5.8 (2.4)	6.0 (3.5–8.0)	7.2 (2.7)^b^	0.015^f^
	Colour teeth (0–10)	5.5 (2.3)	6.0 (4.0–7.0)	6.3 (2.9)^b^	0.131
	Gingiva (0–10)	6.5 (2.1)	7.0 (5.0–8.0)	7.6 (2.4)^b^	0.027^f^
**RAND-36**	Physical functioning (0–100)	85.2 (21.0)	95.0 (72.5–100.0)	90.9 (15.4)^c^	0.224
	Role Limitations due to Physical Problems (0–100)	71.4 (42.8)	100.0 (25.0–100.0)	82.6 (32.8)^c^	0.245
	Bodily Pain (0–100)	71.8 (29.4)	90.0 (45.0–100.0)	74.5 (25.4)^c^	0.679
	General Health (0–100)	67.1 (21.9)	70.0 (50.0–85.0)	78.2 (20.7)^c^	0.060
	Vitality (0–100)	61.2 (21.0)	65.0 (42.5–80.0)	62.1 (20.1)^c^	0.847
	Social Functioning (0–100)	88.1 (18.7)	100.0 (81.3–100.0)	87.7 (20.1)^c^	0.923
	Role Limitations due to Emotional Problems (0–100)	87.3 (31.0)	100.0 (100.0–100.0)	89.0 (26.9)^c^	0.804
	Mental Health (0–100)	85.0 (9.5)	88.0 (84.0–90.0)	80.7 (15.6)^c^	0.055
**QOLS**	Total score (16–112)	91.6 (12.1)	91.0 (83.5–103.0)	83.5 (12.6)^d^	0.006^g^

*Abbreviations*: UCLP: Unilateral complete cleft lip and palate; OHIP-14: The Oral Health Impact Profile-14; OES: The Orofacial Esthetic Scale; RAND-36: The RAND-36-item short health survey; QOLS: The Quality Of Life Scale. ^a^ Dahl et al. (2011)^25^ ; ^b^ Larsson et al. (2014)^27^ ; ^c^ Garratt and Stavem (2017)^31^ ; ^d^ Wahl et al. (2004)^33^ ; ^e^ Welch's t-test; ^f^ p < 0.05; ^g^ p < 0.01.

**Table 2.** Frequency of UCLP Patients Reporting Problems Related to Oral Conditions Given by OHIP-14 Domain and Item Compared to Norwegian Population Norms.

**OHIP-14 Domains**	**OHIP-14 Items**	**UCLP**	**Population Norm Data^a^**
		**Problems^b^ (%)**	**Problems^b^ (%)**
Functional limitations	Trouble pronouncing words	48	7
	Worsened sense of taste	33	7
Physical pain	Painful aching in mouth	48	49
	Uncomfortable to eat food	24	35
Psychological discomfort	Been self-conscious	57	35
	Felt tense	48	25
Physical disability	Unsatisfactory diet	52	8
	Interrupted meals	24	9
Psychological disability	Difficulty relaxing	43	18
	Embarrassed	55	22
Social disability	Irritable with other people	43	8
	Difficulties doing usual jobs	14	7
Handicap	Life less satisfying	29	17
	Unable to function	14	4

Abbreviations: UCLP: Unilateral complete cleft lip and palate; OHIP-14: The Oral Health Impact Profile-14. ^a^ Dahl et al. (2011)^25^ ; ^b^ Problems defined as OHIP-14 score > 0.

The patients with UCLP also rated their orofacial appearance significantly lower than norms, with a UCLP total OES sum score of 40.9 (12.6) as compared to the norm total sum score of 50.3 (15.6) (Table 1). Their lower orofacial rating involved both face, profile, mouth, alignment, and shape of teeth, as well as gingiva.

Overall, the patients with UCLP's generic health as evaluated by RAND-36 were not significantly different from the norm data (Table 1). However, at the domain level, there was a tendency for patients with UCLP to evaluate their perceived mental health better and their perceived general health poorer than the Norwegian general population.

In contrast, the patients with UCLP rated their overall quality of life significantly better than the general population norms, with a UCLP total QOLS sum score of 91.6 (12.1) as compared to the norm total score of 83.5 (12.6) (Table 1). The items demonstrating the highest scores were related to “Satisfaction with spouse and cohabitation” and “Satisfaction with having and rearing children”, whereas the lowest score was related to “Satisfaction with own health” (not shown).

Moreover, the patients with UCLP reported a high level of approach-oriented coping (BACQ-A) and low scores on BACQ-WR and BACQ-D, suggesting little use of avoidant coping strategies (Table 3). The participants’ global self-esteem (RSES) was good, whereas they reported high levels of perceived social support as measured by SPS-24, indicating that they were very satisfied with their social provision (Table 3).

**Table 3.** The UCLP Patients’ Scores on Measures of Personal (BACQ and RSES) and Environmental (SPS-24) Factors Related to Self-Perceived Health and Quality of Life.

Measure	Total Sum Score and Domain Scores (possible score variation)	UCLP mean (SD)	Norm Or Reference Group	
			mean (SD)	p-value^d^
The Brief Approach/Avoidance Coping Questionnaire (BACQ)	Total score (12–60)	45.2 (6.9)	43.5 (7.0)^a^	0.276
	Approach (BACQ-A) (6–30)	23.1 (4.5)	23.5 (3.7)^a^	0.663
	Diversion (BACQ-D) (3–15)	8.2 (2.8)	8.0 (3.2)^a^	0.765
	Withdrawal/Resignation (BACQ-WR) (3–15)	5.7 (2.4)	8.0 (3.2)^a^	< 0.001
Rosenberg Self-Esteem Scale (RSES)	Total score (10–40)	32.8 (5.9)	33.3 (5.5)^b^	0.707
Social Provision Scale (SPS-24)	Total score (24–96)	92.8 (3.2)	77.9 (8.8)^c^	< 0.001

Abbreviations: UCLP: Unilateral complete cleft lip and palate; BACQ: The Brief Approach/Avoidance Coping Questionnaire; RSES: The Rosenberg Self-Esteem Scale; SPS-24: The Social Provision Scale ^a^ Saetre et al. (2025)^35^ ; ^b^ Sinclair et al. (2010)^37^ ; ^c^ Caron et al. (1998)^39^ ; ^d^ Welch's t-test.

Being unemployed was significantly associated with impaired oral-specific health, lower self-perceived orofacial esthetics and lower scores in both physical (physical functioning, role limitations due to physical problems, bodily pain, general health perception) and mental (vitality, role limitations due to emotional problems) health domains of RAND-36, as well as lower overall quality of life (Table 4). Being married or cohabitating was significantly associated with better overall quality of life. No such associations were found for sex, education or the other socio-demographic factors tested (Table 4).

**Table 4.** The Association Between Socio-Demographic Factors and Oral-Specific Health (OHIP-14), Orofacial Esthetics (OES), General Health (RAND-36) and overall quality of life (QOLS) in Patients with UCLP.

	**OHIP-14**	**OES**	**RAND-36**								**QOLS**
	Total score	Total score	Physical Functioning	Role Limitations due to Physical Problems	Bodily Pain	General Health	Vitality	Social Functioning	Role Limitations due to Emotional Problems	Mental Health	Total score
**Sex (male)**	.135	-.326	.295	.400	.382	-.024	.344	.109	.070	-.351	-.215
**Married or cohabitating**	.102	.083	-.186	-.122	.028	.083	.195	.064	.297	.218	.610^b^
**Education**	.166	.233	.084	-.197	-.047	.129	-.131	-.170	-.364	-.158	.188
**Work (no)**	.497^a^	-.444^a^	-.452^a^	-.438^a^	-.454^a^	-.707^c^	-.482^a^	-.420	-.280	-.446^a^	-.593^b^

Abbreviations: UCLP: Unilateral complete cleft lip and palate; OHIP-14: The Oral Health Impact Profile-14; OES: The Orofacial Esthetic Scale; RAND-36: The RAND-36-item short health survey; QOLS: The Quality Of Life Scale. Spearman's rank correlation: ^a^ : p < 0.05; ^b^ : p < 0.01; ^c^ : p < 0.001.

The associations between the patients with UCLP's oral health, orofacial esthetics, generic health or overall quality of life and their coping strategies, self-esteem or perceived social support are given in Table 5. Overall, better oral-specific health (ie, lower OHIP-14 scores) was significantly associated with low BACQ-WR scores and high RSES and SPS-24 scores, respectively. High OES scores were also significantly associated with low BACQ-WR scores, whereas no significant association with self-esteem or social support was demonstrated. The participants’ generic health (RAND-36) domain scores physical functioning, general health perception, vitality, role limitations due to emotional problems and mental health were all significantly negatively associated with their BACQ-WR scores, whereas the RSES scores were significantly associated with the scores of Physical Functioning, Vitality, Role Limitations due to Emotional Problems and Mental Health (Table 5). In addition, better participant overall quality of life as measured by the QOLS sum scores was significantly associated with low BACQ-WR scores and high SPS-24 sum scores, respectively (Table 5).

**Table 5.** The Association Between Self-Perceived Coping (BACQ), Self-Esteem (RSES) or Self-Perceived Provision of Social Support (SPS-24) and Oral-Specific Health (OHIP-14), Orofacial Esthetics (OES), General Health (RAND-36) or Overall Quality of Life (QOLS) in Patients with UCLP.

Measure	OHIP-14	OES	RAND-36								QOLS
	Total score	Total score	Physical Functioning	Role Limitations due to Physical Problems	Bodily Pain	General Health	Vitality	Social Functioning	Role Limitations due to Emotional Problems	Mental Health	Total score
BACQ	-.588^c^	.449^a^	.477^a^	-.288	.229	.472^a^	.418	.369	.315	.447^a^	.472^a^
BACQ-A	-.568^b^	.649^b^	.319	.070	.045	.518^a^	.453^a^	.098	.042	.421	.314
BACQ-D	-.094	.425	-.337	-.273	-.466^a^	-.078	-.116	-.325	-.103	-.309	-.019
BACQ-WR	.762^c^	-.511^a^	-.437^a^	-.331	-.198	-.505^a^	-.472^a^	-.432	-.434^a^	-.602^b^	-.622^b^
RSES	-.579^b^	.316	.490^a^	.348	.151	.289	.486^a^	.300	.517^a^	.497^a^	.327
SPS-24	-.726^b^	.370	.269	.165	.003	.306	.131	.138	.328	.308	.527^a^

Abbreviations: UCLP: Unilateral complete cleft lip and palate; OHIP-14: The Oral Health Impact Profile-14; OES: The Orofacial Esthetic Scale; RAND-36: The RAND-36-item short health survey; QOLS: The Quality Of Life Scale; BACQ: The Brief Approach/Avoidance Coping Questionnaire; BACQ-A: BACQ-Approach; BACQ-D: BACQ-Diversion; BACQ-W/R: BACQ-Withdrawal/Resignation; RSES: The Rosenberg Self-Esteem Scale; SPS-24: The Social Provision Scale. Spearman's rank correlation: ^a^ : p < 0.05; ^b^ : p < 0.01; ^c^ : p < 0.001

## Discussion

Although knowledge of the adult consequences of being born with CL/P is increasing, most investigations so far have focused on the first decade after discharge from multidisciplinary care. In this study of 40-year-old patients with UCLP, the participants reported significant reduced levels of oral-specific health as measured by OHIP-14 and compared to general Norwegian population norms. These findings are consistent with most other studies using OHIP-14 in younger CL/P cohorts.^4,41^ At the domain and item level, significant functional limitations were demonstrated, with problems with pronunciation and difficulties related to eating still being prominent in our patients with UCLP at the age of 40 years. When they were interviewed, participants reported to have articulation problems, fistulas and increased mucus in the upper airways which influenced voice, sometimes causing distress and problems with speaking in public.^5^ Speech problems have been documented to be significantly more common in patients with UCLP than in the general population,^42^ and to be one of the most significant oral problems in CL/P patients in general.^6,9,43^ Moreover, difficulties related to feeding and chewing, the avoidance of certain foods or food textures, as well as worsened taste have also been reported earlier in self-reports from adult CL/P patients.^15,41^ Furthermore, earlier OHIP-14 data have demonstrated that oral-related psychosocial strain may also persist into young adulthood, and a recent report from India studying patients with CL/P and mean age of 22 years at least 2 years after completion of their final treatment described significantly higher scores for embarrassment, irritation and tension in the patients than in the age-matched controls.^44^ This is well in line with our observations and those of Palmeiro et al. in older adults, both showing significantly increased OHIP-14 scores both in the domains Psychological Discomfort and Psychological Disability, revealing self-consciousness, tension, embarrassment, and difficulty relaxing because of problems with their teeth, mouth, or dentures.^41^ Hence, our findings of significantly reduced self-reported oral-specific health in middle-aged patients with UCLP are in line with those earlier demonstrated in younger adults with CL/P and may suggest persisting or unmet needs long after discharge from standardized multidisciplinary care.

Patients’ concerns about appearance are considered an important part of their orofacial health,^27^ and in this study, middle-aged patients with UCLP rated their orofacial esthetics significantly lower than general population norms. Although Lemberger et al. recently reported no significant difference in the OES sum score between 19-year-old patients with UCLP and an age matched control group,^45^ others, using various approaches and measures, have reported that 20%–50% of CLP patients are dissatisfied with aspects of their orofacial appearance when asked in young adulthood.^9,46–48^ Our participants with UCLP rated the esthetics of their face, profile, mouth, alignment, and shape of teeth, as well as their gingiva to be poorer than the norms. Recently, it was reported that 22-year-old patients with CL/P were more satisfied with their dental esthetics than patients at 8 and 12 years of age.^49^ In contrast, Dunworth et al. using the QLEFT-Q and Face-Q instruments found that all esthetic outcomes worsened with age within the age range from 8–22 years.^48^ Consistently, both Marcusson et al. and Oosterkamp et al. studying CL/P patients in their mid-20s found significantly more dissatisfaction with the appearance of nose, lips, mouth, profile and overall facial appearance than groups without clefts.^9,46^ The result of this study demonstrates that still at 40 years of age, patients with UCLP report significant dissatisfaction with their orofacial appearance involving most parts of their mouth and face.

The middle-aged patients with UCLP's perceived generic health status was studied using RAND-36, and for all domains, the score was not statistically different from Norwegian population norms. The participants tended to report that their perceived general health was slightly worse and their mental health slightly better than norms, but the differences were non-significant and smaller than what is usually considered to be of clinical relevance.^29^ Our finding of overall generic health comparable with norms is consistent with several studies of young adults with CL/P.^9,47,50^ However, Sinko et al. found slight reductions of the scores of the domains Social Functioning and Role Limitations due to Emotional Problems,^47^ and others have reported isolated Mental Health domain scores lower than norms in the same age group.^51,52^ Moreover, Mani et al. demonstrated that the subgroup of patients of their cohort who were in their mid-twenties had lower scores of the domains Physical Functioning, Role Limitations due to physical problems, role limitations due to emotional problems, and social functioning when compared to those in their late 30s.^51^ It has been suggested that the first decade after discharge from systematic multidisciplinary care may represent a period of particular challenge for patients with CL/P,^53^ and that improved psychosocial aspects of generic health later in life may be linked to maturation or acceptance.^51^ Our findings that the perceived generic health in middle-aged patients with UCLP was good are supported by the study by Mariano et al. who found that CL/P patients with a mean age of 41 years reported similar or higher domain scores than controls.^54^ In addition, our findings demonstrate that even though the patients with UCLP rated most aspects of their oral-specific health and orofacial esthetics significantly lower than norms, this does not seem to have impacted their perceived generic physical or psychosocial health.

So far, few studies have reported overall quality of life data for adult CL/P patients employing validated instruments, but using the Satisfaction With Life Scale (SWLS), Alighieri et al. and Cochrane and Slade reported overall quality of life similar to controls in cohorts of CL/P patients with mean age of 27 and 33 years, respectively.^16,50^ Although with some variation, our 40-year-old patients with UCLP reported QOLS scores even higher than the Norwegian norms, suggesting good overall quality of life despite their reduced oral-specific health and dissatisfaction with orofacial appearance. This is also consistent with the data obtained by interviewing all our participants the same day as they completed the questionnaire study, where the overall message was that their UCLP had not prevented the participants from achieving goals and having a good life.^5^

The relationships between objective functional and esthetic effects of being born with and treated for CL/P and perceived adult health or overall quality of life are complex, but internal psychological factors like coping style and self-esteem seem to be of importance for individual adjustment.^16,55,56^ Although our cohort of middle-aged patients with UCLP reported poorer perceived oral-specific health and orofacial esthetics, they reported little use of avoidant coping strategies and good global self-esteem compared to norms. In contrast, Cheung et al. found significantly higher social avoidance and lower global self-esteem in Chinese adolescents and adults with CL/P than in a matched group of nonaffected individuals.^55^ They also reported that these differences tended to increase with age. However, most others have reported small or no differences in coping styles and global self-esteem between adolescents and young adults with CL/P and matched controls.^50,57–59^ So far, few studies have addressed possible associations between self-perceived health or quality of life and coping strategies or self-esteem in patients with CL/P. In our patients, low levels of avoidant coping were associated with better perceived oral-specific health, orofacial appearance, generic health, as well as overall quality of life. In addition, higher levels of global self-esteem were associated with better oral-specific and generic health across several domains. When interviewed, our patients also described that having a positive outlook and focusing on strengths may help adjustment and self-acceptance.^5^ Pisula et al. studying a group of CL/P patients aged 16–23 years of age have earlier reported significant negative correlations between the physical and psychological health domains of the questionnaire WHOQOL-BREF and emotional coping strategies, as well as a positive correlation between global self-esteem and overall quality of life.^59^ Although the results of this and other studies^16,59^ are in line with the general holdings of how coping style and self-esteem may influence life satisfaction,^60,61^ and that they are supported by qualitative data provided by CL/P patients,^3,5,56^ further studies are needed to explore these relationships in full depth.

The middle-aged patients with UCLP in this study reported high SPS-24 scores, indicating high levels of perceived social support. In addition, better oral-specific health and overall quality of life were associated with higher levels of social support. In interviews, the value of feeling recognized and receiving support from family and friends during their upbringing have been emphasized by adults born with CL/P.^5,56^ Several studies have also reported that general well-being and positive adjustment in adolescence and adulthood seem to be linked to having supportive relationships with family, friends or significant others including health care providers.^5,56,62,63^ Moreover, Cochrane and Slade found that CL/P patients having close relationships with family members seemed to be more resilient against challenges,^16^ and in this study, being married or cohabitating was significantly associated with better overall quality of life at the age of 40 years. Work may also be seen as a social arena, and there are several reports suggesting that people born with CL/P are likely to be educationally or vocationally disadvantaged.^64,65^ Moreover, having experienced some form of stigma or even bullying at work has been reported by a significant minority of adults with CL/P.^53,66^ Our patients with UCLP did not report more difficulties in doing their usual jobs than norm data. Moreover, data from a large Norwegian population-based registry study suggested that having CL/P did not influence on the socio-economic status.^10^ In addition, adults being born with CL/P have described that having an oral cleft had little influence on them applying for a job or their achievements at work.^9^ Furthermore, qualitative studies have suggested that having CL/P may even help them to stand out in the workplace in a beneficial way.^53^ In this study, unemployment was associated with poorer perceived orofacial appearance, oral-specific and generic health, as well as overall quality of life. Given that being unemployed is almost universally associated with unfavorable physical and mental health outcomes, as well as impaired well-being and risk of social isolation,^67^ it may be of special benefit for CL/P patients to perceive social support and to be included in working life as adults.

In this study, the conceptual model of health-related quality of life described by Wilson and Cleary guided the selection of a comprehensive set of standardized questionnaires that allowed us to demonstrate that at the age of 40 years, our patients with UCLP clearly distinguished between how they perceived their oral specific health, orofacial appearance, generic health, and overall quality of life.^18^ Their reduced oral-specific health and orofacial esthetics did not seem to implicate their perceived generic health or overall quality of life, and moreover, despite their challenges, the overall quality of life was even better than that of the general population norms. This finding may be consistent with adaptation and personal growth, and when interviewed participants described how they had “adjusted to things they had to do” and how they had learnt to “acknowledge own stories”.^5^ Furthermore, they described positive effects of having a cleft, like empathy for others and an urge to give information and reassure other families with a child with CL/P.^5^ Cochrane and Slade have also reported perceptions of positive gains as a result of having a cleft, and that having a positive appraisal of facial appearance was associated with well-being.^16^ This was typically described as change over the course of time or a decrease in the relative importance of the CL/P in the individual's life. Hence, for health care workers, it is on one hand important to acknowledge that most middle-aged adults with CL/P despite their challenges seem to perceive good generic health and satisfaction with life, but on the other hand, that there are substantial individual variation and a significant amount of middle-aged patients who experience orofacial health and appearance challenges that may suggest need for further information, treatment or follow-up. However, in such processes it is essential to value the views of the patients, to see them as individuals and approach their challenges comprehensively.

The small sample size and cross-sectional design represent major limitations of this study, prohibiting multivariate analysis and any causality claims. Moreover, the cohort only included Caucasian Scandinavian participants, possibly reducing the generalizability to other cultures or parts of the world. We also acknowledge that higher levels of education and unemployment in the sample of patients with UCLP may have influenced our results, and that some selection bias may exist as we were unable to include nine potential respondents. On the other hand, the participants in this study represent a unique and well-characterized sample of middle-aged patients with UCLP, operated on by the same surgeon and followed to early adulthood according to a standardized protocol by the same team, reducing possible variations related to cleft type and treatment. It may also be considered a strength that the treatment protocol and the initial data on the participants treatment and follow-up at the age of 5, 16, and 25 years,^19^ as well as interviews at the age of 40 years of age,^5^ have been published earlier. This study adds to the relatively scarce literature on middle-aged patients’ long-time perception of having been born with and treated for CL/P. The use of validated questionnaires based on a theoretical model^18^ allowing for the distinction of oral-specific health, orofacial appearance, generic health and overall quality of life, as well as comparisons with general population norms supplement earlier reports and may represent a strength of this study.

## Conclusion

This study has shown that even at the age of 40 years, participants with UCLP reported variable, but significant cleft-related challenges including reduced oral-specific health and orofacial esthetics compared to norm data. These challenges seem not to have impacted on their generic health or overall quality of life, as these were comparable with or even better than data obtained from the general Norwegian population. Favorable outcomes were associated with less avoidant coping, good self-esteem and good perceived availability of social support, as well as being employed, married or cohabitating. The results supplement interview data obtained from the same participants on the same day as they completed the questionnaires,^5^ allowing for a detailed and nuanced description of how middle-aged patients with UCLP may perceive their health and well-being. The combined results give support to a lifelong and individualized follow-up concept that takes into consideration the experiences and views of the patients and approaches their challenges holistically.

## Data Availability

This project was approved by the Regional Committee for Medical and Health Research Ethics (REC West; p.no. 2016/269). Patient consent for data sharing was not obtained, and participant data will therefore not be shared with third parties.
